# QueryOR: a comprehensive web platform for genetic variant analysis and prioritization

**DOI:** 10.1186/s12859-017-1654-4

**Published:** 2017-04-28

**Authors:** Loris Bertoldi, Claudio Forcato, Nicola Vitulo, Giovanni Birolo, Fabio De Pascale, Erika Feltrin, Riccardo Schiavon, Franca Anglani, Susanna Negrisolo, Alessandra Zanetti, Francesca D’Avanzo, Rosella Tomanin, Georgine Faulkner, Alessandro Vezzi, Giorgio Valle

**Affiliations:** 1 CRIBI Biotechnology Centre, University of Padua, Padua, Italy; 2 Department of Biology, University of Padua, Padua, Italy; 3 Department of Medicine, University of Padua, Padua, Italy; 4 Department of Women’s and Children’s Health, University of Padua, Padua, Italy; 5 Present address: Department of Biotechnology, University of Verona, Verona, Italy

**Keywords:** Variant prioritization, Exome sequencing, Variant annotation, Data integration

## Abstract

**Background:**

Whole genome and exome sequencing are contributing to the extraordinary progress in the study of human genetic variants. In this fast developing field, appropriate and easily accessible tools are required to facilitate data analysis.

**Results:**

Here we describe QueryOR, a web platform suitable for searching among known candidate genes as well as for finding novel gene-disease associations. QueryOR combines several innovative features that make it comprehensive, flexible and easy to use. Instead of being designed on specific datasets, it works on a general XML schema specifying formats and criteria of each data source. Thanks to this flexibility, new criteria can be easily added for future expansion. Currently, up to 70 user-selectable criteria are available, including a wide range of gene and variant features. Moreover, rather than progressively discarding variants taking one criterion at a time, the prioritization is achieved by a global positive selection process that considers all transcript isoforms, thus producing reliable results. QueryOR is easy to use and its intuitive interface allows to handle different kinds of inheritance as well as features related to sharing variants in different patients. QueryOR is suitable for investigating single patients, families or cohorts.

**Conclusions:**

QueryOR is a comprehensive and flexible web platform eligible for an easy user-driven variant prioritization. It is freely available for academic institutions at http://queryor.cribi.unipd.it/.

**Electronic supplementary material:**

The online version of this article (doi:10.1186/s12859-017-1654-4) contains supplementary material, which is available to authorized users.

## Background

Over the past few years, the advances in DNA sequencing technology have opened new perspectives in many fields of Life Sciences. In particular, Whole Genome Sequencing (WGS) and Whole Exome Sequencing (WES) are contributing to the extraordinary progress in the study of genetic variants, improving the understanding of causative genes in human disorders.

While “Next Generation Sequencing” (NGS) is making the production of sequencing data progressively easier, bioinformatic analysis is still a problem when dealing with genes and pathologies not well characterized at the molecular level.

The initial bioinformatic steps for variant analysis are quite standard: the NGS reads are firstly aligned on the human reference genome [1], then the resulting SAM file [2] is parsed for the identification of genomic variants. As a result, a Variant Call Format (VCF) file with the list of variants is generated [3].

The selection of candidate variants responsible for the phenotype or disease under study remains a challenging task. Firstly, we need to functionally characterize and annotate the large number of variants that are typically detected: tens of thousands for WES and millions for WGS. Several approaches have been developed to accomplish this task. Programs like SIFT [4] and PolyPhen-2 [5] evaluate variants by focusing on the impact of amino acid changes on protein function, while ANNOVAR [6] extends the functional annotation considering other features such as phylogenetically conserved regions and allele frequency in populations.

Once the variants have been annotated further action is required to choose the most effective criteria for “prioritizing” candidate causative variants. It is unfeasible to conceive an all-purpose protocol as the type of problems and the available data may be very disparate. Moreover, field-specific expertise may be essential both in the definition of the criteria and in the interpretation of the data.

If the genetic disease is well characterized at the molecular level, then the obvious action to take is to focus on the variants occurring on known causative genes. Unfortunately, our knowledge is still limited as ~50% of Mendelian monogenic diseases have not yet been associated with causative genes [7], while most polygenic disorders remain uncharacterized at the molecular level.

Taking into consideration that the function of many genes is still unknown, bioinformatic approaches such as Endeavour [8] prioritize candidate genes on features shared with other genes that are involved in the same biological process or disease under study. Several phenotype-driven approaches have been implemented in programs like eXtasy [9], PhenIX [10], Phenolyzer [11], PHIVE [12], Exomiser [13] and Phevor [14], taking advantage of resources such as Gene Ontology (GO) [15], Human Phenotype Ontology (HPO) [16] and Disease Ontology (DO) [17].

As previously mentioned, the prioritization process usually requires the integration of a wide range of functional information about variants, genes and diseases as well as mode of inheritance when multiple individuals are considered. Currently, the standard strategy involves the application of filters with arbitrary thresholds that progressively remove variants not satisfying the criteria. As a result there is the risk of removing something that is just below the threshold for one of the criteria, while being well above the threshold for the other criteria.

Prioritization is not only confined to the problem of merging information on variants, genes and phenotypes. An issue that is often disregarded is that the vast majority of genes undergo alternative splicing [18]. As a result the same variant may have very different functional outcomes, for instance it may generate a stop codon in a transcript and a silent variant in another isoform of the same gene. For this reason the annotation of variants should refer to each alternative transcript rather than the putative major isoform.

Recently, some web-servers [19] have been developed to analyze exome data, but they do not satisfy most of the above requirements, thus limiting the spectrum of possible analyses. Stand-alone programs such as VariantMaster are available [20], but they are driven by line-commands that make their usage cumbersome and difficult for most users. An additional problem is that our knowledge on human genomics is changing very rapidly at all levels, needing continuous updates, implementations and integration of data, tools and ideas. Therefore, a platform for prioritization that combines usability and comprehensiveness has become a priority.

With these premises in mind, QueryOR has been engineered as a user friendly web-platform that integrates the most advanced prioritization criteria. Furthermore, QueryOR is built on a robust set of XML-defined rules that allows an easy implementation of new criteria without modifying the program code. Currently, 70 different criteria of prioritization have been implemented in the platform and can be selected by users to build dynamic tailor-made queries and to facilitate expert-driven variant and gene prioritization.

## Implementation

### Web-interface implementation

QueryOR has been implemented in CGI/Perl combined with Apache web-server. JavaScript, Jquery, AJAX and CSS properties are used to dynamically render some parts of HTML pages and to define their structures and layouts. The pages for criteria selection and transcript report are built on dedicated XML-files. For this reason, we have developed a XML-language that describes standard database queries and their web representation (layout, form elements, hyperlinks, highlighted columns). Thus, any selection criterion or transcript data table is completely specified in a XML node, making the system flexible and scalable. The XML language also allows the user to integrate custom databases into the QueryOR platform. This integration is easily obtained loading multicolumn files with information related to genes (one column must contain the ENSEMBL gene ID) or variants (four columns are mandatory: chromosome, position, reference allele and alternative allele). Once the file is loaded, the user can select the fields on which one or more filters have to be created. Then, the system automatically fills a new database associated to the project and builds specific XML-files containing the new queries, which will be available with all other criteria.

### Data processing implementation

The data processing step is based on in-house scripts developed in Perl, Python and Bash; it runs on a blade cluster, managed by a PBS job resource manager (TORQUE). ANNOVAR software and dbNSFP database (v2.9) [21] are used for the annotation of variants, in addition to a homemade script. All project data are stored in a local database using MariaDB, a MySQL open-source fork, with the TokuDB® engine. The database is designed to contain both annotation tables and user data tables. The former host human gene annotations and known SNP information (global minor allele frequency, clinical significance, etc.) and are regularly updated every 6 months. The latter stores the data uploaded by the user and the associated meta-data produced during the “Data processing” step.

### ENSEMBL data and variant annotation integration

The hg19 release 81 of human gene and transcript data has been downloaded from ENSEMBL (http://grch37.ensembl.org/info/data/ftp/) [22]. Two different databases of known mutations have been integrated in the platform: dbSNP [23] version 144 (http://www.ncbi.nlm.nih.gov/SNP/) [24], modified to recover old variants excluded from this last release but present in the online version, and Exome Variant Server version ESP6500SI-V2 (http://evs.gs.washington.edu/EVS/) [25] have been chosen to annotate allelic frequencies in the population. Disease information has been obtained from OMIM (http://www.omim.org/) [26] and associated to gene and transcript data. Regarding somatic mutations, QueryOR incorporates COSMIC database [27] version 74, whose SQL table has been created starting from VCF files containing both coding and non-coding mutations and the complete export file of COSMIC. In case of new releases of gene annotations, dbSNP files or OMIM data, a custom set of Perl/Python scripts have been developed for the automatic update of all SQL tables.

### Integration of functional and phenotypic annotations

QueryOR integrates several gene annotations derived from different public databases, which have been directly obtained from their respective websites or through ENSEMBL BioMart [28]. Within these annotations, QueryOR embeds Gene Ontology [29] and InterPro [30] data, as well as two different pathway repositories, KEGG (Kyoto Encyclopedia of Genes and Genomes) [31] and Reactome [32], which have been collected using the Graphite package [33] of Bioconductor [34]. QueryOR also makes available gene expression data derived from the GTEx portal (version 6) [35]. The information contained in this atlas has been processed to link Ensembl ID to tissues and sub-tissues in which the gene is expressed. The level of expression is measured in RPKM (Reads Per Kilobase per Million mapped reads) [36]. Moreover, regarding the phenotype annotation, the platform accommodates two main databases: DisGeNET version 3.0 [37] and Human Phenotype Ontology (HPO) release 98, whose entries have been further processed to be associated to ENSEMBL-ID. The updating of these functional annotations has been automatized through a set of Perl/Python scripts as described in the previous section.

### Chromosome map tool implementation

The “runs of homozygosity” (ROHs) are calculated by comparing the user-uploaded variants and the high-polymorphic dbSNP variants (GMAF higher than 0.3) falling into the target regions. The algorithm extracts those positions where only dbSNP variants, and no custom variants, are mapped. The resulting locations are those with a homozygous genotype for the reference allele (0/0) in the analyzed sample.

Using these spots, the script finds all the ROHs, computes the length distribution and selects the stretches whose length exceeds the 95th percentile of the distribution. Then, the algorithm tries to extend all the ROH seeds in both directions as long as the homozygosity ratio (number of positions with 0/0 genotype divided by the sum of homozygous and heterozygous positions in the considered region) remains above 0.9. ROHs are used to build the “chromosome map” chart in association with the genes selected during the prioritization process.

### Case study dataset

The exome data from the “Diagnostic Exome Sequencing in Persons with Severe Intellectual Disability” (study EGAS00001000287, https://www.ebi.ac.uk/ega/studies/EGAS00001000287) [38, 39] were obtained from the European Genome-Phenome Archive (EGA) web site.

## Results

**Fig. 1:**
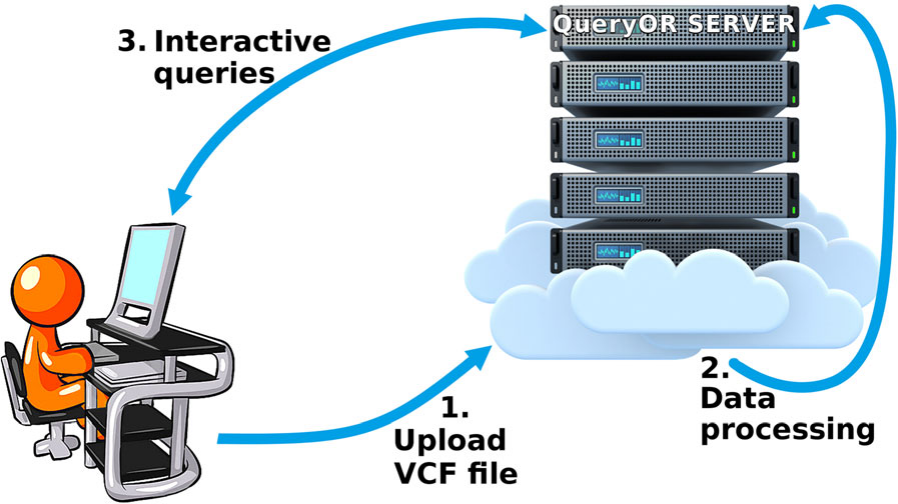
The three main steps of QueryOR analysis. Step 1 and step 3 require interaction with the user, whereas step 2, data processing, is automatically performed by the system after uploading VCF files

We have implemented QueryOR dividing the process into three main steps as shown in Fig. 1.

Each step is further divided into different sub-steps and procedures, as detailed below. Users will spend most of their time at step 3, querying and browsing the system in the search of possible causative variants. To test the potential and features of the querying step, several sets of data have been made openly available on the platform, including some trio data from de Ligt et al. [38], as well as data produced by our own group.

### Uploading and updating VCF files

All QueryOR’s activities are centered on projects that the users can create and possibly share with their collaborators. Projects can be related to single individuals, trios or families, as well as population or cohorts. Starting a project is very simple, but users must first register, both for privacy reasons and for permitting the retrieval of their data.

The creation of a project requires the uploading of VCF files that must satisfy several requirements. Firstly, each individual sample should be labeled with a unique name that will be used as identifier in the subsequent steps. Secondly, the information about genotype, allelic depth and total read depth, which are usually found in the GT, AD and DP fields, must be available. Although VCF is a well established format, not all variant callers implement the VCF fields in the same way; for instance the Torrent Variant Caller does not fill the AD and DP fields. Therefore, we have developed specific scripts that calculate the allelic and total read depth from other parameters, such as Alternate allele Observations (AO) and Reference allele Observation count (RO). As a result, the platform accepts VCF files produced by all the commonly used variant callers.

**Fig. 2:**
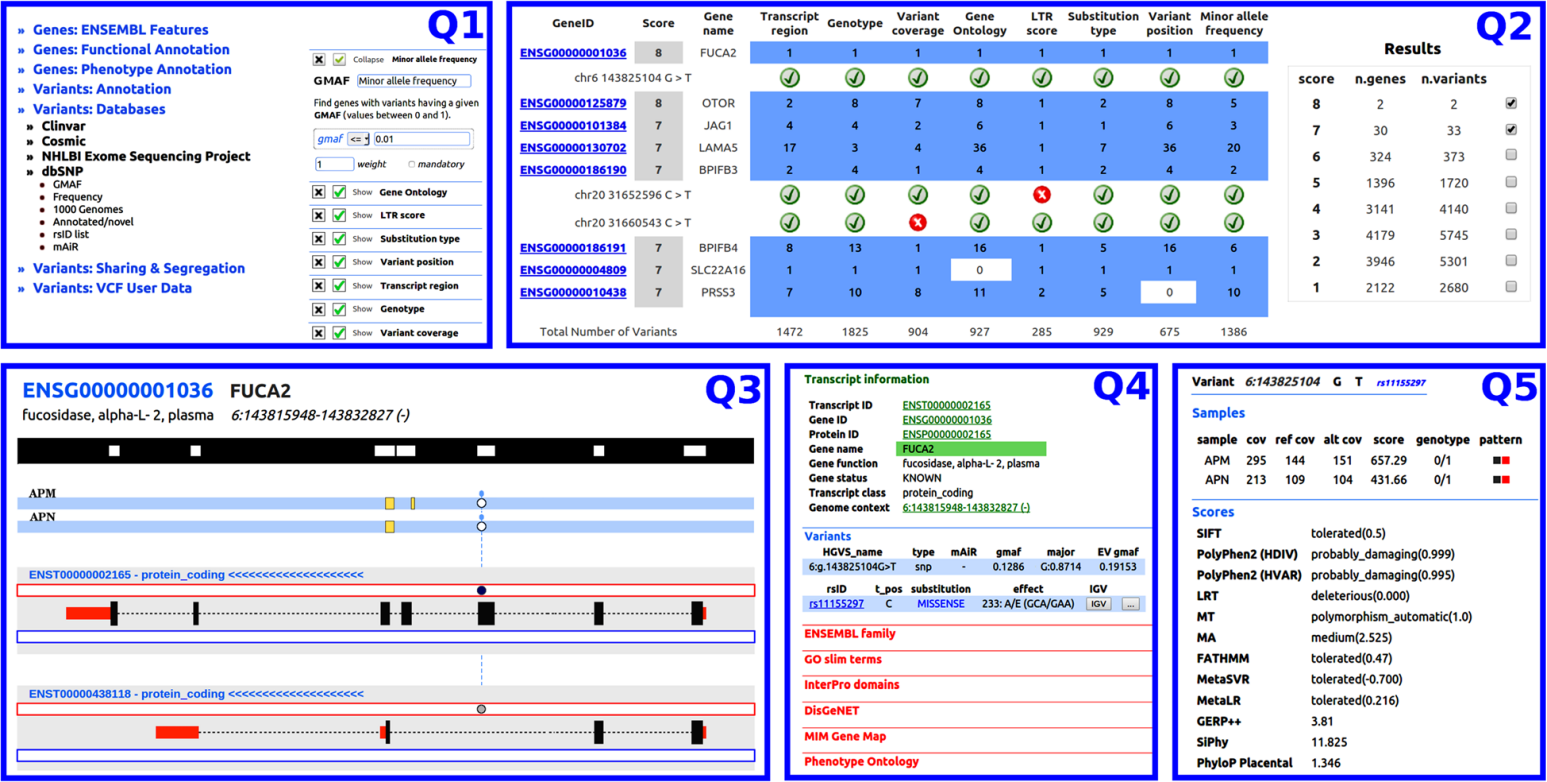
A typical route for a QueryOR investigation starts with the selection of criteria (Q1); a ranked list of genes and variants is returned in Q2. The selection of a gene, for instance FUCA2, leads to page Q3 where variants and affected transcripts in their genomic context are shown. The *black* track at the top of Q3 shows the target regions of exome capturing. The blue tracks just below show that the analysis was done on two samples named APN and APM, that share a heterozygous variant (*white circles*). The *yellow boxes* report the positions specified in the optionally uploaded BED file, indicating for instance low coverage regions. The bottom part of Q3 shows two alternative transcripts where the same variant in one case is located in an exon, generating a missense substitution (*dark blue circle*) while in the other case is located in an intron (*gray circle*). By clicking on a transcript of Q3, the system returns Q4, where several transcript features are directly linked to external resources, as well as to the variant overview page (Q5). For a full list of symbols used in Q3, see Fig. 3. A more detailed description of the entire process is given in the main text

In the upload/update step the user can also upload BED files containing regions of interest. BED files should have four columns for each row: chromosome number, starting position, ending position and sample ID; the latter is used to associate the genomic coordinates to the right individual. These custom-defined regions will be shown in the graphical synopsis of variants and transcripts (Fig. 2-Q3) as yellow boxes. We usually exploit this feature to mark on each sample the regions with low coverage.

Once the files are uploaded, QueryOR takes some time, from minutes to hours, to process data, depending on the number of uploaded samples and variants. The user can check the job status while the processing is running. The beginning and the end of the process are notified by automatic emails to the user’s registered address.

### Data processing

Data are processed by an automatic back-end procedure that provides a comprehensive annotation of the variants, linking them to genes, transcripts, encoded proteins and biological ontologies. QueryOR takes into consideration that alternative splicing may generate multiple transcripts from the same gene. As a result, a variant may have different effects depending on the transcript isoform. With this premise, we thought that the common practice of limiting variant annotation to the major transcript isoform is a coarse approximation. Therefore, to manage this problem QueryOR annotates variants on all the predicted ENSEMBL transcripts derived from alternative splicing events. Furthermore, the distribution of variants on the different splicing isoforms can be displayed and examined by the user as a part of the interactive result analysis described in the next paragraph.

Besides QueryOR’s own procedures, a further double annotation is performed using both ANNOVAR [6] and dbNSFP [21], thus obtaining a wide set of measures, scores and constraints related to each variant, that among others include SIFT [4], PolyPhen [5], MutationAssessor [40] and GERP++ [41].

Data processing involves many other steps, including the association of variants to the available information in dbSNP, such as the allelic frequency in the global population and in ethnic groups, as well as the presence in the 1000 Human Genome Project [42]. Moreover, we discovered several thousand SNPs in the reference genome (both in GRCh37 and GRCh38) that do not correspond to the major allele in the population and as a consequence are found as “false positive” in most individuals. To overcome this problem, the reference positions characterized by a dbSNP frequency lower than 0.1 are annotated as mAiRs (minor Allele in Reference).

When a project involves the analysis of multiple patients such as trios and families, the platform runs a specific module that automatically computes how variants are shared between individuals. Moreover, possible Runs of Homozygosity are calculated for each sample, as explained in the Methods section.

All the retrieved and computed information obtained by the data processing step is stored in the QueryOR database.

The overall time required for loading and processing data is approximately proportional to the number of variants. Typically, for ~100,000 unique variants (6-8 exomes) the time required is less than 20 min. A more detailed analysis of the loading time is given in Additional file 1: Figure S1.

### Interactive queries and results analysis

After the completion of data processing, the user can explore the information that has been associated to the project, following the general procedure shown in Fig. 2. Queries can be formulated very easily and the resulting answers are typically delivered in a few seconds that can extend to minutes for very complex queries. Thus it is possible to experiment different criteria and parameters, to perform a comprehensive investigation and to get progressively closer to possible causative genes. A detailed analysis of the querying time, as a function of the number of criteria and variants can be found in Additional file 1: Figure S2.

The complete route from query to variant takes five progressive steps that correspond to pages appearing on the web browser, labelled Q1 to Q5. At each step some decisions must be taken: Q1 is for the query, Q2 is for choosing a gene from the resulting list, Q3 is for the selection of a specific transcript among the different isoforms, Q4 corresponds to the transcript report where a certain variant can be chosen and Q5 is the description of the variant. Like being in a maze, you may explore some paths and you can go back if the route leads to a dead end. In the web browser, Q1 to Q5 will open as independent pages making it easy to return to any of the previous steps. Some integrated QueryOR tools are associated to different points of this route, to make decisions easier. The main features of this process are described in the following paragraphs.

#### Query procedure (Q1)

Page Q1 allows the user to select the criteria for prioritization that are grouped into seven main sections. Three sections (ENSEMBL Features, Functional Annotation and Phenotype Annotation) are related to genes, pathways and phenotypes. In these sections it is possible to select for specific lists of genes and transcripts as well as features like gene ontology, gene expression and associations to pathways, diseases or phenotypes. The remaining four sections are related to variants. These include Variants Annotation (for instance genomic context and functional prediction scores), Variants Databases (for instance dbSNP, EVS and COSMIC), Variants Sharing and Segregation (variants in homozygosity and/or heterozygosity present or absent in different individuals) and VCF User Data (for instance variant coverage, genotype and quality calls).

Each section can be exploded to visualize sub-sections that can be further expanded to see the selectable criteria. Figure 2-Q1 shows a query page where the section Variants Databases shows four sub-sections and where the last sub-section (dbSNP) shows six selectable criteria. The selected criteria are shown on the right side of frame Q1 where GMAF is under definition, while other 7 defined criteria are shown in their “collapsed” view.

By default all criteria have the same relevance in the ranking process, but this can be modified by assigning different weights to each criterion. There are no restrictions in the number of selected criteria, but very complex queries may take a longer processing time.

#### Engine (Q2)

When a query is submitted, the system performs an independent search for each of the selected criteria; then, the score of each variant is calculated as the sum of the weights of the satisfied criteria. Finally, genes are ranked according to their highest-score variant. The results from the query are summarized in a score table (right part of Fig. 2-Q2) that shows the number of genes and variants associated to each score. The two top-scores shown in the right side of Fig. 2-Q2 were selected and expanded to produce the results matrix on the left, where each row reports a single gene combined with the number of variants satisfying the prioritization criteria.

By clicking on a gene name in the results matrix, more details show up. For instance, the image in Fig. 2-Q2 was taken after expanding *FUCA2* and *BPIFB3*. This feature is useful to better understand the results. In fact, although the first six genes have positive variants in every column, as shown by the blue background, only 2 genes satisfy all the 8 selected criteria, resulting in an associated score of 8. This apparent incongruence can be explained by looking at the expanded data of *BPIFB3*, showing that although the gene has some variants satisfying all the criteria, the two best variants satisfy only 7 criteria.

From the bottom line of Q2 (Total Number of Variants) it is possible to appreciate the depth and the stringency of each filter and to make a general evaluation of the prioritization. Thus the user can reconsider some of the criteria and go back to Q1 to redefine the query.

#### Gene overview (Q3)

This page is shown after a gene is selected by clicking on the Gene-ID, in the results matrix. The page displays a compact graphical representation of alternative transcripts associated to the selected gene, together with the position and type of each variant across all samples. In Fig. 2-Q3, two samples named APM and APN are shown at the top of the frame. Both samples share a heterozygous variant, represented by the white dots. The bottom part of the Q3 frame shows two alternative transcripts in which the same variant acts as a missense mutation (dark blue dot) in one transcript and as an intronic mutation (gray dot) in the other.

**Fig. 3:**
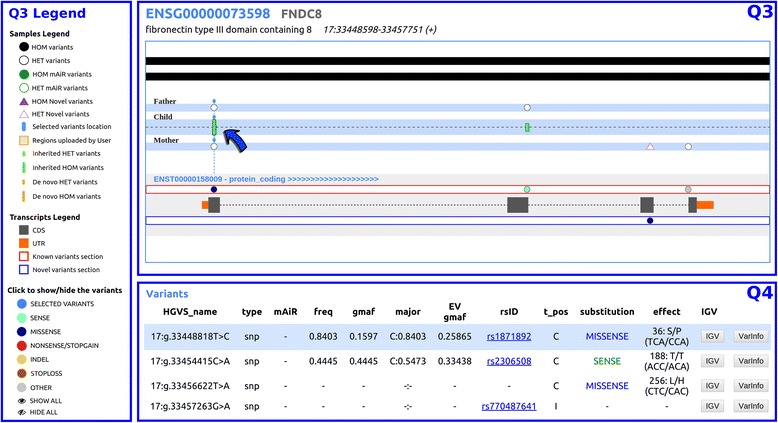
Trio analysis. In the Q3 section, the arrow points to a variant that is heterozygous in both parents and homozygous in the child (*full green bar*). At the end of the next exon, the child displays a heterozygous variant, shown as a small *green bar*, which was directly inherited from the father. A detailed description of the variants is given in the Q4 section where the user can also find a link to the IGV viewer, that will be conveniently opened on the appropriate genomic position

In the case of trio studies, samples are differently tracked to highlight parental heritage of allelic variants (haplotype configuration), as shown in Fig. 3.

#### Transcript report (Q4)

Detailed information about the transcript selected in Q3 is shown in Q4 (Figs. 2 and 3), where various contents are briefly described and directly linked to their primary source on the web. The variants that emerged from the prioritization process are highlighted with a blue background. If the BAM file is available on the client side, the user can consider to launch IGV [43] that will automatically point to the position of the variant under analysis to view the alignment of the reads on the genome. By the “Varinfo” button the user can move to Q5.

#### Variant overview (Q5)

This page allows the evaluation of the specific features of the candidate variant (Fig. 2-Q5) where several pathogenicity scores are accessible, including the above mentioned PolyPhen and SIFT, as well as Mutation Taster [44], CADD [45] and DANN [46]. Although these features are sometimes discordant, it is useful to have a global view to estimate the possible pathogenicity of the variant under analysis.

#### Advanced analyses

From page Q2 it is possible to access other QueryOR’s tools such as the “Variants Report” that is a printable table summarizing the information on variants, genes and pathogenicity. Another link builds a “Chromosome map” reporting possible Runs Of Homozygosity, that can be important in the analysis of human disorders, as they represent a good clue for the presence of deleterious variants responsible for recessive diseases [47]. A further link takes the user to the “Gene Analysis tool” that allows the identification of genes carrying different mutations among a group of patients. With this tool it is possible to investigate unrelated patients or to investigate diseases caused by *de novo* mutations, where it is more important to know if the same gene is mutated in different patients rather than if they share the same variant. This information comes as a summary table flanked by a distribution chart (data not shown). Each group of genes can be further investigated searching for shared biological terms, using DAVID [48], or for common pathways within Reactome [32] and KEGG [31].

### Case study

To evaluate the performance of the platform we re-analyzed some of the data published by de Ligt et al. [38], (EGA study EGAS00001000287), concerning patients affected by recessive forms of cognitive impairment and mental retardation. Our prioritization strategy was achieved by applying several criteria on trio number 4 (VCF files EGAZ00001004509, EGAZ00001004510, EGAZ00001004511). In particular: 1) we selected high confidence variants with coverage level >60 and 2) with alternative allele coverage >30; 3) we only considered variants that changed the amino acid sequence; 4) as the disease is rare, we imposed a low frequency threshold with maf < 0.05; 5) the results were further fine-tuned by considering the “intellectual disability” Phenotype Ontology keyword; 6) taking into consideration the pattern of inheritance, we selected variants that are homozygous only in the child. QueryOR identified only two variants that could satisfy these six criteria. Interestingly, one of the two is a missense variant placed in the PDHA1 gene, in the X chromosome, corresponding to that proposed in the aforementioned work [38]. It is interesting to point out that with only six criteria it was possible to achieve a very effective prioritization. The above case is fully explained in a tutorial available at http://queryor.cribi.unipd.it/cgi-bin/queryor/tutorial.pl. To prevent any incidental findings and to preserve patients privacy, the tutorial is based on the exome of a healthy patient, manually edited to insert the above variant.

## Discussion

It is normal that when a new technology starts to produce novel types of data, the development of software analysis runs a little behind and eventually catches up. In the case of Whole Genome and Exome Sequencing this problem is particularly relevant because the scope of the prioritization process is not limited to the variants as such, but it extends also to a wide variety of data and information that is continuously updated and is often superseded by new discoveries.

When we started the development of QueryOR, this context of generalized “work in progress” was one of our main concerns. Prioritization is essentially a process of data integration and to develop it using unstable datasets would be a vain effort. On the other hand, we thought that a user friendly variant-prioritization platform, suitable for a wide range of analyses, could be of great utility. To overcome the problem of sustainability, QueryOR has been designed on a general schema rather than on predefined databases. A dedicated XML language permits the declaration of the datasets to be implemented in the platform. Each dataset is defined for its content, for the possible queries and for their web representation (layout, form elements, hyperlinks, highlighted columns), thus making the system flexible and scalable.

Thanks to this flexibility many datasets are available in the platform while more will be added in the future. Although a query could be potentially made by selecting different features from all the available datasets, in a normal session only some of the data will be interrogated. Thus there is a double level in which the information is organized: at the basal level there are all the available datasets implemented by the QueryOR manager, while at the top there is the information emerging from the queries performed by the end-users.

**Fig. 4:**
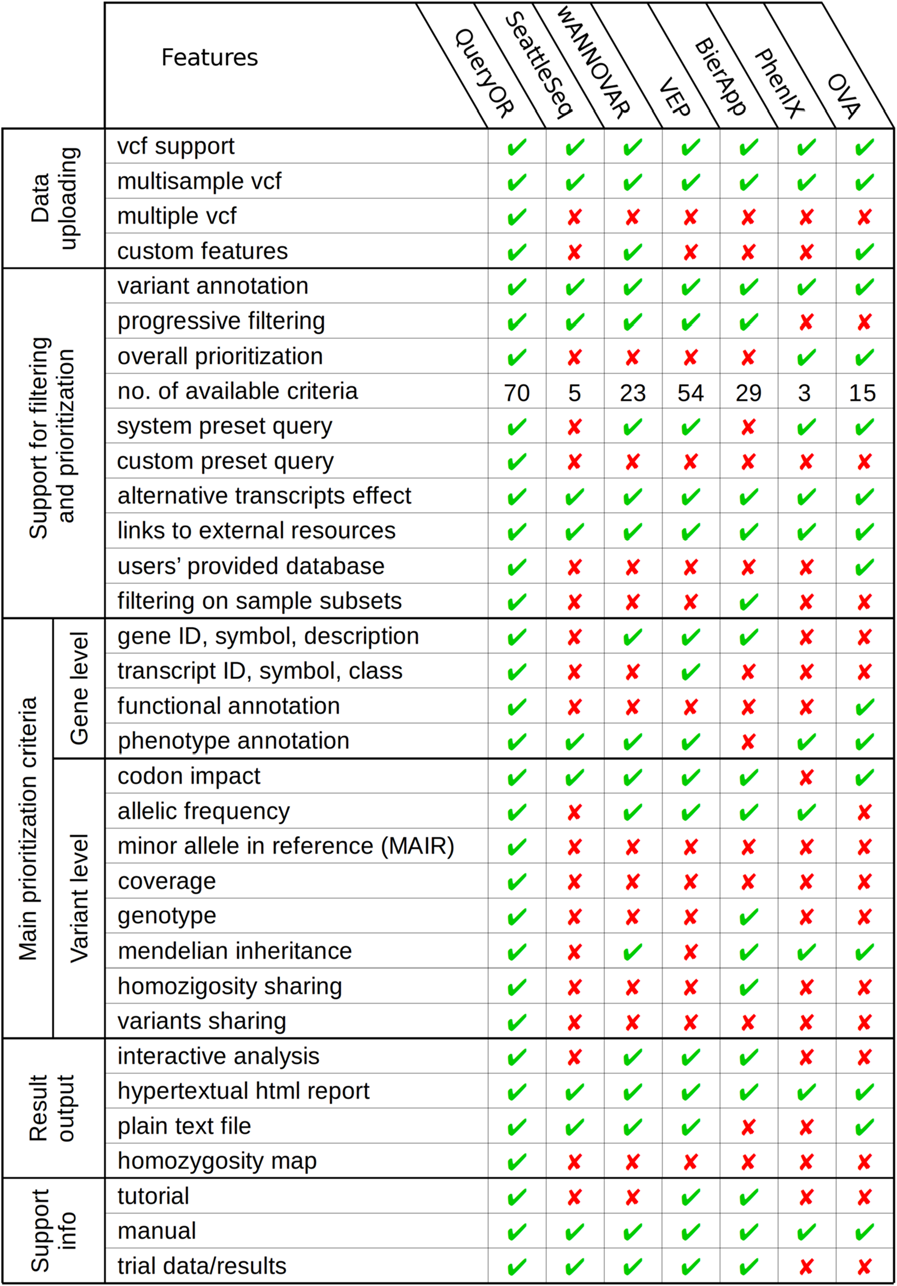
Comparison of QueryOR with other platforms for variant prioritization. The platforms were tested using a VCF input file. The indicated number of available criteria is approximate due to different ways of implementation

In literature, several bioinformatic tools for whole exome analysis are reported, but only a few of them are suitable for a comprehensive and efficient exome investigation. In fact, while some platforms center their analyses on gene features found in biological ontologies, others focus primarily on variant annotations, disregarding gene function. In QueryOR we combined the most useful features found in other tools, gathering and expanding them within a single platform. Moreover, to enhance the potential of the analyses, we implemented some important features such as the annotation of minor alleles in the reference genome, several prioritization criteria based on VCF information such as coverage, genotype and quality score, as well as criteria based on sharing variants and homozygosity in different individuals. Furthermore, we introduced the possibility to implement customized prioritization criteria based on databases supplied by the user. A detailed description of the procedure for submitting custom tables is given in the User Manual, available in the “Info” section of the web site. Figure 4 compares the main features of QueryOR with other available tools, including SeattleSeq [49], wANNOVAR [6], VEP [50], BierApp [19], PhenIX [10] and OVA [51].

To our knowledge, QueryOR is the open web tool with the widest spectrum of applicable criteria (currently 70) for exome data prioritization, spanning from gene and variant annotations, to intrinsic features of the VCF file. Another interesting peculiarity of QueryOR regards the opportunity to select a subset of samples within a multisample project, allowing focusing on attributes found only in the chosen group of samples.

**Fig. 5:**
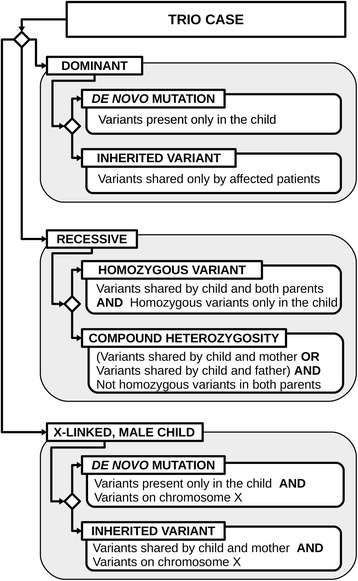
Usage of the criteria for “shared” and “homozygous” variants in a trio case. Diamonds indicate different hypotheses that can be made. For instance, if we hypothesize a recessive homozygous variant in the child we should set two criteria: 1) shared variants by child and both parents and 2) homozygous variants only in the child. Whereas, for a compound heterozygosity we would expect that the child shares the variants, but we do not know which variant is in which parent; furthermore, the variant should not be homozygous in the parents. Compound heterozygosities are generally difficult to find and criteria based only on sharing and homozygosity would not be selective enough. In this case the “Gene Analysis tool” described in the text could help in the selection of genes carrying different mutations. Sometimes it may be useful to set criteria that may appear useless, like homozygosity on a X chromosome; however this may help to reduce false positives

A major effort has been made to simplify the formulation of complex queries. To perform a query the user can select any combination of criteria and associated parameters. For instance, one of the criteria could be the minimal coverage of the locus where a SNP occurs and the associated parameter could be “30”. Criteria can be classified in three main categories. The first group is based on the knowledge of genes and diseases, exploiting the functional and phenotypical annotation integrated in QueryOR as well as lists of candidate disease genes when available. The second category discriminates variants on the basis of information contained in the VCF file including coverage, genotype and quality of calling. The third category is related to variant features, such as pathogenicity scores, effect on protein, population frequency and distribution among the project samples. In particular, it is possible to impose a specific inheritance model in trios as schematized in Fig. 5, or families and cohorts, allowing for instance the selection of variants shared or not shared among different patients or that are homozygous in some patients and heterozygous in others.

**Fig. 6:**
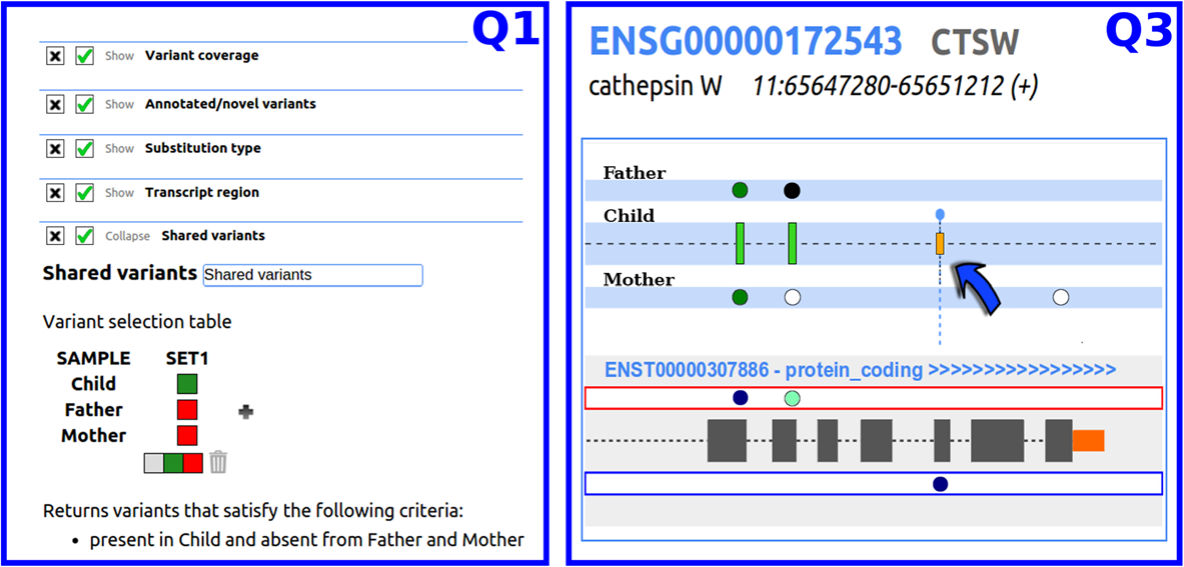
Searching for *de novo* mutations in a trio. Q1: to set the criteria the user should select “Shared variants” and click the box beside each patient, selecting *green*, *red* or *gray* respectively for present, absent and ignore, while the sentence underneath will report in plain English the meaning of the settings; more criteria can be set by clicking the + symbol. Q3: the results include haplotype phasing. The *yellow bar* indicated by the arrow is a possible *de novo* mutation. For the meaning of other symbols see Fig. 3

In the development of the graphical user interface, we dedicated a particular attention to user friendliness, both for setting the criteria and for interpreting the results. As an example, Fig. 6 shows how *de novo* mutations can be searched and visualized in a trio of mother, father and child.

In contrast with other similar tools that return only the items that simultaneously satisfy all the query specifications, QueryOR sorts the results on the number and weight of satisfied criteria; thus, the user can have a global view of which criteria are or are not met for every gene and can decide whether to continue the investigation or modify the query. The integration of a wide range of heterogeneous information and the automated annotation procedure provides the end user with the ability to evaluate the information at various levels in order to establish the relationships between different data and to discriminate between causal and neutral variants.

Several other innovative features of QueryOR make the process of prioritization thorough and at the same time easy. For instance, an important issue is that we annotated all the variants that in the reference genome are represented by rare alleles, that we named mAiRs (minor Allele in Reference). These variants can either be filtered off by the query specification or alternatively they will be automatically labelled as mAiR when seen on the selected genes.

## Conclusion

Currently, QueryOR is primarily used to analyse exomes and gene panels, however it has been successfully employed also for whole genomes. In this respect the main problem is the lack of functional information that can be associated to variants belonging to non-coding sequences. As this information will become available we will take advantage of the flexibility of QueryOR to implement datasets that may facilitate the prioritization of variants in whole genome analyses.

In conclusion, the comprehensiveness of the implemented criteria and the aptness to add new features together with a user-friendly environment make QueryOR very suitable to support researchers, clinicians and geneticists engaged in variant analyses.

## Availability and requirements

Project name: QueryOR

Platform home page: http://queryor.cribi.unipd.it

Tutorial: http://queryor.cribi.unipd.it/cgi-bin/queryor/tutorial.pl

User manual: http://queryor.cribi.unipd.it/cgi-bin/queryor/user_manual.pl

Access requirements: Web browser

Access restrictions: None

## Additional file

Additional file 1: This file contains supplementary figures supporting the manuscript. **Figure S1** time required for uploading and processing a project. **Figure S2** time required for the processing of a query. (ODT 121 kb)

## Abbreviations

AD: Allele depth
AO: Alternate Allele observations
BAM: Binary alignment map
BED: Binary extended data
DO: Disease ontology
DP: Filtered depth
GMAF: Global minor Allele frequency
GO: Gene ontology
GT: Genotype codes
HPO: Human phenotype ontology
mAiR: Minor Allele in reference
NGS: Next generation sequencing
RO: Reference Allele observation count
ROH: Runs of homozygosity
RPKM: Reads per kilobase per million mapped reads
SQL: Structured query language
VCF: Variant call format
WES: Whole exome sequencing
WGS: Whole genome sequencing
XML: eXtensible markup language
