# Supplementary material for: QueryOR: a comprehensive web platform for genetic variant analysis and prioritization
Source: BMC Bioinformatics. 2017 Apr 28;18:225. doi: 10.1186/s12859-017-1654-4 (PMC5410040; doi:10.1186/s12859-017-1654-4)
Supplement: Supplementary file 1 [file 12859_2017_1654_MOESM1_ESM.odt]

Additional file 1

Figure S1.  Time required for uploading and processing a project.   The Figure shows the loading and processing time of about 200 projects, as a function of their number of unique variants. It can be seen that the required time is roughly proportional to the number of unique variants; however some discordance may be due to different ratios of novel/known variants, as well as to resource availability on the computer cluster.

Figure S2.  Time required for the processing of a query. The Figure shows the time required for the processing of queries with different number of criteria and with increasing number of variants. All the tests were repeated three times and the figure shows the mean and standard deviation of each point.
